# Dr. Cappe, on Cow-Pox

**Published:** 1801-04

**Authors:** R. Cappe

**Affiliations:** York


### *To the Editors of the Medical and Phyſical Journal.*

Gentlemen,

As the preſervation of Vaccine matter is important, for conveying it to diſtant countries, and for ſecuring a ſteady progreſs of the new inoculation in remote parts of our own country, where freſh Vaccine matter cannot be frequently procured; I am inclined to believe you will not think the following fact unworthy of your notice, eſpecially as Mr. Hill, in the laſt number of your Journal, ſpoke of the means of preſerving Vaccine matter, and gave preference to a method leſs to be approved than that which I have adopted. He miſtook what I mentioned incidentally in a provincial news-paper. I recommended, that the matter be incloſed between two pieces of glaſs, folded in paper. It is thus more perfectly ſecluded from air than when put into a phial. In this way I firſt received Vaccine matter from Dr. Jenner, as active as when freſh and fluid. I have lately preferred putting the two pieces of glaſs together, while the matter on them is ſtill moiſt, by which means all air is excluded, except from the margin, and that, if it be thought injured, may eaſily be removed when the matter is uſed.

I have for many months intended to make ſucceſſive inoculations with Vaccine matter of advancing age, to aſcertain how long it may be preſerved; but I have not hitherto executed my purpoſe. One experiment, however, ſhews that it may be preſerved long enough to be conveyed to very diſtant countries. On the 11th of February, David Collier, a year and half old, was inoculated with Vaccine matter taken on the 23d of October, and preſerved in the manner deſcribed. He has taken the diſeaſe, which now appears in a perfectly regular form, although he has twice ſcratched off the ſurface of the riſing veſicle. In this inſtance, the matter, ſixteen weeks old, ſeemed in no reſpect leſs active than the fluid taken immediately from a recent veſicle.

The preſervation of Vaccine matter might be more certain, if the edges of the glaſſes put together were bruſhed over with ſpirit varniſh, or ſealing-wax diſſolved in ſpirit of wine: If the two pieces of glaſs were of unequal ſize, the larger would leave a margin, on which the varniſh lodging might form a perfect ſeal againſt air and water. Matter thus ſecured might probably be defended againſt the injury of hot climates, by being folded in oiled ſilk, and put in jars full of water, to prevent it being injured by motion; and theſe jars, when occaſion required, required, might be kept cool by being plunged in veſſels of water, often repleniſhed, and ſuffered to evaporate.

It is much to be wiſhed, that the bleſſing of Dr. Jenner's great diſcovery were imparted to our diſtant colonies: As ſome attempts to convey Vaccine matter to the Weſt Indies have failed, any means that offer a chance of conveying it ſafe, deſerve attention. If eaſier methods fail, it may probably be conveyed to any place, however diſtant, by inoculating cows or human beings in ſucceſſion during the voyage.

In temperate weather, Vaccine matter may be kept fluid many hours, if it be inſtantly encloſed between two pieces of glaſs. But I think no advantage can be derived from preſerving it fluid, if that be poſſible for any conſiderable time, ſince in a dry ſtate it is leſs likely to ſuffer chemical change. It becomes hard, not merely by evaporation of moiſture; it ſeems to coagulate; for when evaporation is impeded, it ſtill grows thick; and the dried matter does not form a perfect ſolution in water.

While writing on the ſubject, allow me to tranſcribe a paragraph from that letter in the news-paper to which Mr. Hill alluded. I wiſh to know whether my ſuſpicion be confirmed by the obſervation of any other perſon. "In four inſtances, in which inoculation was made with matter dried on lancets, a bit of thread imbued with matter from ſome other patient was placed on the inciſion: In one, the Cow-pox had the regular courſe; in the three others, it was ſo different in the earlier ſtages, that I entertain a doubt whether thoſe patients be ſecure againſt the ſmall-pox, and ſhall inoculate them again. Theſe facts ſuggeſt the precaution, not to inoculate with matter from two patients at once. If future experience do not juſtify this caution, it will, in the mean-time, be attended with no inconvenience." This was written on the 10th of October; ſince that time, care has been taken, that a lancet uſed in puncturing one veſicle, was well waſhed before it was uſed for another, and that matter from two patients was in no way blended. I have ſince ſeen no irregular caſes, when I had not reaſon to believe that the uſual progreſs of inoculation was diſturbed by violence done to the inciſion; and none in which any troubleſome ulceration occurred, though that happened to two of the three patients mentioned in the paragraph juſt quoted.

The inference Dr. Watkinſon drew from the facts ſtated in his book, and from the bills of mortality quoted by Mr. Franks, do not merit unreſerved aſſent; but I know not whether it be worth while to enter farther into this diſcuſſion; however, I will not at preſent intrude longer on your patience. I am, &c.

*York, Feb.* 21, 1801.

R. CAPPE.

